# Is Genetic Mobilization Considered When Using Bacteriophages in Antimicrobial Therapy?

**DOI:** 10.3390/antibiotics6040032

**Published:** 2017-12-05

**Authors:** Lorena Rodríguez-Rubio, Joan Jofre, Maite Muniesa

**Affiliations:** Department of Genetics, Microbiology and Statistics, Faculty of Biology, Av. Diagonal 643, 08028 Barcelona, Spain; lorenarodriguez@ub.edu (L.R.-R.); jjofre@ub.edu (J.J.)

**Keywords:** bacteriophages, antimicrobials, lysins, horizontal gene transfer, transduction

## Abstract

The emergence of multi-drug resistant bacteria has undermined our capacity to control bacterial infectious diseases. Measures needed to tackle this problem include controlling the spread of antibiotic resistance, designing new antibiotics, and encouraging the use of alternative therapies. Phage therapy seems to be a feasible alternative to antibiotics, although there are still some concerns and legal issues to overcome before it can be implemented on a large scale. Here we highlight some of those concerns, especially those related to the ability of bacteriophages to transport bacterial DNA and, in particular, antibiotic resistance genes.

## 1. Revival of Phage Therapy

The World Health Organization (WHO) has identified antibiotic resistance as one of the most challenging problems in public health care on a global scale [1]. The Centers for Disease Control (CDC) estimate that each year around 2 million people in the USA suffer from bacterial infections caused by antibiotic-resistant bacteria, with at least 23,000 resulting in death [2]. In the European Union, antimicrobial resistance is responsible for 25,000 deaths per year and costs around 1.5 billion EUR in healthcare and productivity losses [3]. The report launched by the WHO on 20 September, 2017 [4] shows that as of May 2017, only 8 out of 51 antibiotics in the clinical pipeline belong to new classes, indicating a serious lack of novel antibiotic development. The urgent need for alternative or complementary therapies to control bacterial infections has prompted the rediscovery of phage therapy (the use of bacterial viruses to treat bacterial infections).

As the most abundant entities on Earth, with approximately 10^31^ viral particles [5], bacteriophages, or phages, play a crucial role in the regulation of bacterial populations. After their discovery in the second decade of the 20th century, the capacity of phages to kill pathogenic bacteria led to their therapeutic application against infectious diseases. However, during the early trials of phage therapy (for an extensive review, see [6]), several mistakes were made, mostly attributed to insufficient knowledge about the biological nature of phages. Low titers, preparations contaminated with bacterial antigens, or phages with no infectivity for the bacterial target were used [7]. As a result, the success rate of phage treatment was not constant and after the introduction of antibiotics it was abandoned as unreliable in many parts of the world, with the exception of the Soviet Union and Eastern Europe [8].

The increase of pathogens with multiple resistances to antibiotics has revived interest in phage therapy. Thus, several studies using animal models have been performed against clinically relevant pathogens such as *Pseudomonas aeruginosa*, *Clostridium difficile*, Vancomycin-resistant *Enterococcus faecium*, β-lactamase-producing *Escherichia coli*, *Acinetobacter baumannii*, or *Staphylococcus aureus*, where bacteriophages were used to treat bacteremia or sepsis with a good rate of success in reducing mortality [7]. The phages were mostly administered by intraperitoneal injection, although subcutaneous injection and oral administration were also used in some cases. Clinical trials have also been performed, mostly against antibiotic-resistant *S. aureus* and *P. aeruginosa* (for an extensive review, see [6]). However, these have been primarily focused on safety rather than efficacy [9], since safety concerns are still a major hurdle for the development of phage therapy. 

Phages are ubiquitous: both humans and animals carry many different types and are in constant contact with them, so it is reasonable to assume they are not harmful. Although to date no phage products have been approved for human therapy in the USA or EU, several phage cocktails are “Generally Recognized as Safe” (GRAS) products by the US Food and Drug Administration (www.fda.gov) and approved for use in the food industry [10]. On the other hand, the more conservative European Food Safety Authority (EFSA) awarded phages a “Qualified Presumption of Safety” (QPS) in 2007. Recent reports issued by the EFSA on the use of phages in food production [11] indicate that, in the opinion of the EFSA scientific panel of experts, phages have great potential, but further research is advisable for each specific phage application. Most of the reports describing the in vitro efficacy of phages state their usefulness for a variety of antimicrobial applications, although phage–bacteria interactions in an active infection and the involvement of the immune system are difficult to reproduce in vitro [12].

## 2. Advantages and Potential Disadvantages of Phage Therapy

Many claims have been made about the advantages of phage therapy, notably the highly specific manner in which phages target their host bacterial strain. In addition, phages display great diversity and are relatively easy to isolate. Once the host is infected, phage propagation leads to host lysis at the end of the lytic cycle and the release of virion progeny. The resulting exponential growth in phage numbers amplifies the treatment and the possibilities of success. Phages are also self-limiting, multiplying only as long as host bacteria are present, and they have an inherent low toxicity, since they consist exclusively of proteins and DNA [13]. As clinical trials show, phages are effective against antibiotic-resistant pathogens [6], indicating a lack of cross-resistance with antibiotics. 

Opponents of phage therapy always point out the rapid emergence of phage-resistant bacterial mutants and the adverse reaction of the immune system against the phage. However, some strategies to avoid these potential drawbacks have been devised. The most common solution to avoid resistance involves using a phage cocktail, instead of a single phage, since the host is unlikely to become resistant to all the phages simultaneously. Moreover, the emergence of resistant mutants is also a risk of antibiotic treatment, but has never been a reason to discard it. The immune system response could be avoided or minimized by selecting phages with characteristics that are unlikely to trigger an immune response. Interestingly, the immune system does not always thwart phage therapy efficacy. Roach and Leung [14] showed that synergy between the immune system and bacteriophages is essential for the success of phage therapy in the treatment of pulmonary infections caused by *P. aeruginosa*.

## 3. Bacteriophages and Horizontal Gene Transfer

In our opinion, there is, however, another important issue that is not usually taken into account when selecting phages for therapeutic application. Phages are responsible for a considerable amount of horizontal gene transfer and the evolution of their genomes is characterized by an unusually high degree of horizontal genetic exchange [15]. During the lytic cycle, bacterial rather than phage DNA may be packaged into the phage capsid, producing a transducing particle that upon release from the (donor) host cell can transfer this bacterial DNA to another (recipient) cell [16]. The fact that phages can mobilize bacterial DNA means they can also mobilize and transduce virulence genes [17,18,19], antibiotic resistance genes [20,21,22,23,24], or genes related to fitness [25,26]. Although transduction was previously thought to be the consequence of errors in the phage packaging machinery and therefore a rare event, occurring approximately once every 10^7^–10^9^ phage infections [27], recent data suggest this is not the case, as the ratio of transducing particles to lytic phages varies upon prophage induction by different agents and conditions, including antibiotics [28].

By avoiding temperate phages, this problem could certainly be minimized, although not completely resolved. Quite a large number of virulent phages are also capable of mobilizing bacterial sequences, either via generalized transduction events [29,30,31,32] or other mechanisms still not completely understood [33]. Phages regulate their own induction and mobilize themselves, but they can also be hijacked by non-self-mobile elements that use phage capsids to spread. Examples can be found in the *Staphylococcus* genera [34,35,36]. Different parts of the bacterial genome are mobilized, and to date it is not clear if this occurs randomly or is orchestrated to favor the transfer of specific genes. It seems reasonable that the transfer of genes related to the virulence, survival, or fitness of the host strain—such as antibiotic resistance genes (ARGs)—will be favorably selected [19,21,23,37,38]. Moreover, a certain proportion of recombination events (homologous or illegitimate) are responsible for the mosaic structure of phages. These events can take place once phage DNA (even from a virulent phage) reaches the inner cell, and they can occur either between phage and bacterial DNA, or between phage and prophage DNA, as prophage-containing cells are common in most bacterial groups [39,40]. Though quite unpredictable, such events have been observed to take place during generalized transduction [41,42,43]. The consequences of this recombination could be an additional problem for the inclusion of phages in food or medicines [44], since new recombinant phages could be generated. All these gene transfer mechanisms and recombination events have been reported quite recently, and others will probably appear in the light of new findings resulting from the complete sequencing of bacterial genomes.

## 4. Bacteriophages as ARG Mobilizing Elements

Despite the growing challenge of antibiotic resistance, we know surprisingly little about how ARGs are transferred between strains, species, and even genera, and even less about how environments and gene-expression levels influence transmission. It seems likely that the use of antibiotics and other antimicrobials increases selective pressure on bacteria that carry ARGs and the vectors that mobilize these genes. From these bacteria, ARGs can be horizontally transferred to other bacteria by mobile genetic elements, most commonly plasmids and transposons, although recently it has been proposed that bacteriophages are also involved [20,45,46]. Bacteriophages basically consist of one nucleic acid molecule (the phage genome) surrounded by a protein coating, the capsid. The packaging of the nucleic acid in a protein capsid confers protection and hence extracellular persistence, which cannot be found in naked DNA or RNA. Since phage-packaged DNA is protected from degradation, and phages can persist in different extracellular environments without losing their infectious capabilities, gene transfer by transduction might be more important than previously thought. Some bacterial genera can produce phage-like elements using information encoded in their own genome. These particles, called gene transfer agents (GTAs), have a bacteriophage-like capsid and although so far they have been reported exclusively in α-proteobacteria [33,47,48], it is reasonable to expect that these mechanisms, or similar ones, could play a role in the spread of bacterial DNA in other bacterial groups. While there is no evidence for the exact origin of GTA genes, there is no doubt they are identical to phage genes.

A recent study has reported that bacteriophages (understood as complete phage particles containing phage DNA) rarely encode ARGs [49], suggesting that instead bacterial DNA is packaged in phage particles. This is supported by our own and other authors’ findings that ARGs occur in the bacteriophage DNA fraction of human fecal samples [45], hospital wastewater [50], aquaculture wastewater [51], sludge [52], raw wastewater [53,54], and environmental samples [20]. Providing further evidence, Lekunberri et al. [55] analyzed 33 viromes of different origins and found that while human-associated viromes rarely encode ARGs, the non-human viromes contain a large ARG reservoir, suggesting that bacteriophages or phage particles could play an important role in the spread of resistances in the environment. 

It has also become clear that phages play a role in the fine-tuning of all known microbiomes [56]; even their influence on the homeostasis of the microbiota and welfare of the individual cannot be excluded [57,58,59]. In light of this evidence, we should consider the implications of introducing a cocktail of up to 10^9^ PFU/mL of phages into a fine-tuned microbiome, even if they have been confirmed as strictly virulent. The potential impact of this introduction on microbiomes, including gene mobility, is unpredictable. A lack of caution and careful planning when antibiotics first came into use has led to the resistance crisis we are now facing.

## 5. Phage Lytic Proteins: A Suitable Alternative in Phage Therapy to Avoid the Risk of Genetic Transfer

As phage therapy is one of the most feasible alternatives to antibiotics and due to all the aforementioned concerns, phage lytic enzymes have also been explored as antimicrobials. There are two general classes of phage lytic proteins that mediate the enzymatic cleavage of peptidoglycans (PGs): endolysins and virion-associated peptidoglycan hydrolases (VAPGHs) [60,61]. While VAPGHs degrade PGs in the first stages of phage infection prior to phage DNA injection, endolysins are expressed in the last stages, ensuring the release of the phage progeny via bacterial lysis. The potential of these phage lytic enzymes as antimicrobials and biotechnological tools has been extensively reviewed (see [60,61,62,63]), thus here we only point out their applicational advantages over the complete phage particle. 

Due to their high specificity and strong activity, bacteriophage lytic enzymes may be as effective as phages while offering additional benefits. Since they are incapable of transferring genetic content, the potential problems of horizontal gene transfer and recombination events are avoided. As enzymes cannot propagate as phages do, their effect is limited to the first doses, allowing better control of a possible influence on microbiota homeostasis. Like phage particles, phage lytic proteins have already proven their efficacy in vitro as well as in animal models [64]. Regarding human applications, Phase I and II clinical trials have been completed by GangaGen Inc. (http://www.gangagen.com, Palo Alto, CA, USA) for the intra-nasal use of an anti-staphylococcal phage protein, and ContraFect (http://www.contrafect.com, Yonkers, NY, USA) is carrying out Phase II trials for the intravenous use of CF-301 to treat *S. aureus* bacteremia (ClinicalTrials.gov Identifier NCT03163446), whose results have not yet been published. For topical application, Staphefekt™ developed by Micreos (http://www.micreos.com, Bilthoven, The Netherlands) is the first endolysin approved for use in humans on intact skin. It is commercialized under the brand Gladskin, which includes products to treat *S. aureus* skin infections (www.gladskin.com, Micreos Human Health, The Hague, The Netherlands).

Phage lysins possess many properties that make them suitable as therapeutics. Their effectivity at low doses would reduce both the immune response and therapy costs, and they display synergistic effects with other antimicrobials [63]. They are highly specific, destroying the target pathogen without affecting commensal microflora. Due to their proteinaceous nature, they are noncorrosive and biodegradable. Several studies have reported the development of antibodies against endolysins upon systemic or mucosal application in animal models [63], but no adverse effects or anaphylaxis were observed and no inactivation by antibodies occurred. Moreover, one of the most interesting features of phage lytic proteins is that no resistance to these enzymes has been reported so far, despite attempts to find it [63,65].

The therapeutic potential of phage lytic proteins has prompted the development of tailor-made antimicrobials based on these enzymes. Their unique modular structure enables domain shuffling, giving rise to antimicrobials with the desired specificity and enhanced activity [64]. Initially, the major disadvantage of phage lytic enzymes as antimicrobials was their inefficacy against Gram-negative bacteria due to the latter’s outer membrane barrier. However, this issue seems to have been resolved with the development of Artilysin^®^ engineered enzymes that combine the lytic activity of a phage-derived enzyme with the outer membrane-penetrating activity of an antimicrobial peptide [66,67].

In our opinion, the therapeutic use of phage lytic proteins is more feasible and advisable than that of complete infective phage particles. The limitations to be expected are similar to those of many other therapeutic products: the engineering and production of the enzymes, which require previous optimization.

## References

[1] WHO (2013). Antibiotic Resistance—A Threat to Global Health Security.

[2] CDC (2013). Antibiotic Resistance Threats in the United States.

[3] European Commission Antimicrobial Resistance. https://ec.europa.eu/health/amr/antimicrobial-resistance_en.

[4] WHO (2017). Antibacterial Agents in Clinical Development—An Analysis of the Antibacterial Clinical Development Pipeline, Including Tuberculosis.

[5] Suttle C.A. (2005). Viruses in the sea. Nature.

[6] Abedon S.T. (2017). Bacteriophage Clinical Use as Antibacterial “Drugs”: Utility and Precedent. Microbiol. Spectr..

[7] Lin D.M., Koskella B., Lin H.C. (2017). Phage therapy: An alternative to antibiotics in the age of multi-drug resistance. World J. Gastrointest. Pharmacol. Ther..

[8] Abedon S.T., Kuhl S.J., Blasdel B.G., Kutter E.M. (2011). Phage treatment of human infections. Bacteriophage.

[9] Vandenheuvel D., Lavigne R., Brüssow H. (2015). Bacteriophage Therapy: Advances in Formulation Strategies and Human Clinical Trials. Annu. Rev. Virol..

[10] (2006). GRAS Notice Inventory—Agency Response Letter GRAS Notice No. GRN 000198.

[11] European Food Safety Authority (2009). The use and mode of action of bacteriophages in food production. EFSA J..

[12] Reindel R., Fiore C.R. (2017). Phage Therapy: Considerations and Challenges for Development. Clin. Infect. Dis..

[13] Stephen T., Abedon A.J.C. (2012). Phage Therapy: Emergent Property Pharmacology. J. Bioanal. Biomed..

[14] Roach D.R., Leung C.Y., Henry M., Morello E., Singh D., Di Santo J.P., Weitz J.S., Debarbieux L. (2017). Synergy between the Host Immune System and Bacteriophage Is Essential for Successful Phage Therapy against an Acute Respiratory Pathogen. Cell Host Microbe.

[15] Hatfull G.F. (2008). Bacteriophage genomics. Curr. Opin. Microbiol..

[16] Zinder N.D. (1995). Bacterial transduction. J. Cell. Comp. Physiol..

[17] O’Brien A., Newland J., Miller S., Holmes R., Smith H., Formal S. (1984). Shiga-like toxin-converting phages from Escherichia coli strains that cause hemorrhagic colitis or infantile diarrhea. Science.

[18] Allué-Guardia A., García-Aljaro C., Muniesa M. (2011). Bacteriophage-encoding cytolethal distending toxin type V gene induced from nonclinical *Escherichia coli* isolates. Infect. Immun..

[19] Penadés J.R., Chen J., Quiles-Puchalt N., Carpena N., Novick R.P. (2015). Bacteriophage-mediated spread of bacterial virulence genes. Curr. Opin. Microbiol..

[20] Colomer-Lluch M., Jofre J., Muniesa M. (2011). Antibiotic resistance genes in the bacteriophage DNA fraction of environmental samples. PLoS ONE.

[21] Haaber J., Leisner J.J., Cohn M.T., Catalan-Moreno A., Nielsen J.B., Westh H., Penadés J.R., Ingmer H. (2016). Bacterial viruses enable their host to acquire antibiotic resistance genes from neighbouring cells. Nat. Commun..

[22] Muniesa M., García A., Miró E., Mirelis B., Prats G., Jofre J., Navarro F. (2004). Bacteriophages and diffusion of beta-lactamase genes. Emerg. Infect. Dis..

[23] Ross J., Topp E. (2015). Abundance of antibiotic resistance genes in bacteriophage following soil fertilization with dairy manure or municipal biosolids, and evidence for potential transduction. Appl. Environ. Microbiol..

[24] Colavecchio A., Cadieux B., Lo A., Goodridge L.D. (2017). Bacteriophages contribute to the spread of antibiotic resistance genes among foodborne pathogens of the *Enterobacteriaceae* family—A review. Front. Microbiol..

[25] Lindell D., Sullivan M.B., Johnson Z.I., Tolonen A.C., Rohwer F., Chisholm S.W. (2004). Transfer of photosynthesis genes to and from *Prochlorococcus* viruses. Proc. Natl. Acad. Sci. USA.

[26] Müller M.G., Ing J.Y., Cheng M.K.-W., Flitter B.A., Moe G.R. (2013). Identification of a phage-encoded Ig-binding protein from invasive Neisseria meningitidis. J. Immunol..

[27] Bushman F. (2002). Lateral DNA Transfer. Mechanisms and Consequences.

[28] Stanczak-Mrozek K.I., Laing K.G., Lindsay J.A. (2017). Resistance gene transfer: Induction of transducing phage by sub-inhibitory concentrations of antimicrobials is not correlated to induction of lytic phage. J. Antimicrob. Chemother..

[29] Petty N.K., Toribio A.L., Goulding D., Foulds I., Thomson N., Dougan G., Salmond G.P.C. (2007). A generalized transducing phage for the murine pathogen *Citrobacter rodentium*. Microbiology.

[30] Ripp S., Ogunseitan O.A., Miller R.V. (1994). Transduction of a freshwater microbial community by a new *Pseudomonas aeruginosa* generalized transducing phage, UT1. Mol. Ecol..

[31] Lee S., Kriakov J., Vilcheze C., Dai Z., Hatfull G.F., Jacobs W.R. (2004). Bxz1, a new generalized transducing phage for mycobacteria. FEMS Microbiol. Lett..

[32] Monson R., Foulds I., Foweraker J., Welch M., Salmond G.P.C. (2011). The *Pseudomonas aeruginosa* generalized transducing phage φPA3 is a new member of the φKZ-like group of “jumbo” phages, and infects model laboratory strains and clinical isolates from cystic fibrosis patients. Microbiology.

[33] Stanton T.B. (2007). Prophage-like gene transfer agents-novel mechanisms of gene exchange for *Methanococcus, Desulfovibrio, Brachyspira*, and *Rhodobacter* species. Anaerobe.

[34] Quiles-Puchalt N., Carpena N., Alonso J.C., Novick R.P., Marina A., Penadés J.R. (2014). Staphylococcal pathogenicity island DNA packaging system involving cos-site packaging and phage-encoded HNH endonucleases. Proc. Natl. Acad. Sci. USA.

[35] Novick R.P., Christie G.E., Penadés J.R. (2010). The phage-related chromosomal islands of Gram-positive bacteria. Nat. Rev. Microbiol..

[36] Frígols B., Quiles-Puchalt N., Mir-Sanchis I., Donderis J., Elena S.F., Buckling A., Novick R.P., Marina A., Penadés J.R. (2015). Virus Satellites Drive Viral Evolution and Ecology. PLoS Genet..

[37] Brown-Jaque M., Calero-Caceres W., Muniesa M. (2015). Transfer of antibiotic-resistance genes via phage-related mobile elements. Plasmid.

[38] Von Wintersdorff C.J.H., Penders J., Van Niekerk J.M., Mills N.D., Majumder S., Van Alphen L.B., Savelkoul P.H.M., Wolffs P.F.G. (2016). Dissemination of antimicrobial resistance in microbial ecosystems through horizontal gene transfer. Front. Microbiol..

[39] Fortier L.-C., Sekulovic O. (2013). Importance of prophages to evolution and virulence of bacterial pathogens. Virulence.

[40] Casjens S. (2003). Prophages and bacterial genomics: What have we learned so far?. Mol. Microbiol..

[41] Chiura H.X. (1997). Generalized gene transfer by virus-like particules from marine bacteria. Aquat. Microb. Ecol..

[42] Thierauf A., Perez G., Maloy A.S. (2009). Generalized transduction. Methods Mol. Biol..

[43] Beumer A., Robinson J.B. (2005). A broad-host-range, generalized transducing phage (SN-T) acquires 16S rRNA genes from different genera of bacteria. Appl. Environ. Microbiol..

[44] Brüssow H., Canchaya C., Hardt W.-D. (2004). Phages and the evolution of bacterial pathogens: From genomic rearrangements to lysogenic conversion. Microbiol. Mol. Biol. Rev..

[45] Quirós P., Colomer-Lluch M., Martínez-Castillo A., Miró E., Argente M., Jofre J., Navarro F., Muniesa M. (2014). Antibiotic resistance genes in the bacteriophage DNA fraction of human fecal samples. Antimicrob. Agents Chemother..

[46] Balcazar J.L. (2014). Bacteriophages as Vehicles for Antibiotic Resistance Genes in the Environment. PLoS Pathog..

[47] Zhao Y., Wang K., Budinoff C., Buchan A., Lang A., Jiao N., Chen F. (2009). Gene transfer agent (GTA) genes reveal diverse and dynamic *Roseobacter* and *Rhodobacter* populations in the Chesapeake Bay. ISME J..

[48] Lang A.S., Zhaxybayeva O., Beatty J.T. (2012). Gene transfer agents: Phage-like elements of genetic exchange. Nat. Rev. Microbiol..

[49] Enault F., Briet A., Bouteille L., Roux S., Sullivan M.B., Petit M.-A. (2017). Phages rarely encode antibiotic resistance genes: A cautionary tale for virome analyses. ISME J..

[50] Subirats J., Sànchez-Melsió A., Borrego C.M., Balcázar J.L., Simonet P. (2016). Metagenomic analysis reveals that bacteriophages are reservoirs of antibiotic resistance genes. Int. J. Antimicrob. Agents.

[51] Colombo S., Arioli S., Guglielmetti S., Lunelli F., Mora D. (2016). Virome-associated antibiotic-resistance genes in an experimental aquaculture facility. FEMS Microbiol. Ecol..

[52] Calero-Cáceres W., Melgarejo A., Colomer-Lluch M., Stoll C., Lucena F., Jofre J., Muniesa M. (2014). Sludge as a potential important source of antibiotic resistance genes in both the bacterial and bacteriophage fractions. Environ. Sci. Technol..

[53] Calero-Cáceres W., Muniesa M. (2016). Persistence of naturally occurring antibiotic resistance genes in the bacteria and bacteriophage fractions of wastewater. Water Res..

[54] Colomer-Lluch M., Calero-Cáceres W., Jebri S., Hmaied F., Muniesa M., Jofre J. (2014). Antibiotic resistance genes in bacterial and bacteriophage fractions of Tunisian and Spanish wastewaters as markers to compare the antibiotic resistance patterns in each population. Environ. Int..

[55] Lekunberri I., Subirats J., Borrego C.M., Balcázar J.L. (2017). Exploring the contribution of bacteriophages to antibiotic resistance. Environ. Pollut..

[56] Virgin H.W. (2014). The virome in mammalian physiology and disease. Cell.

[57] Navarro F., Muniesa M. (2017). Phages in the human body. Front. Microbiol..

[58] Norman J.M., Handley S.A., Baldridge M.T., Droit L., Liu C.Y., Keller B.C., Kambal A., Monaco C.L., Zhao G., Fleshner P. (2015). Disease-Specific Alterations in the Enteric Virome in Inflammatory Bowel Disease. Cell.

[59] Pérez-Brocal V., García-López R., Nos P., Beltrán B., Moret I., Moya A. (2015). Metagenomic Analysis of Crohn’s Disease Patients Identifies Changes in the Virome and Microbiome Related to Disease Status and Therapy, and Detects Potential Interactions and Biomarkers. Inflamm. Bowel Dis..

[60] Nelson D.C., Schmelcher M., Rodriguez-Rubio L., Klumpp J., Pritchard D.G., Dong S., Donovan D.M. (2012). Endolysins as Antimicrobials. Adv. Virus Res..

[61] Rodríguez-Rubio L., Martínez B., Donovan D.M., Rodríguez A., García P. (2013). Bacteriophage virion-associated peptidoglycan hydrolases: Potential new enzybiotics. Crit. Rev. Microbiol..

[62] Rodríguez-Rubio L., Gutiérrez D., Donovan D.M., Martínez B., Rodríguez A., García P. (2016). Phage lytic proteins: Biotechnological applications beyond clinical antimicrobials. Crit. Rev. Biotechnol..

[63] Schmelcher M., Donovan D.M., Loessner M.J. (2012). Bacteriophage endolysins as novel antimicrobials. Future Microbiol..

[64] Roach D.R., Donovan D.M. (2015). Antimicrobial bacteriophage-derived proteins and therapeutic applications. Bacteriophage.

[65] Rodriguez-Rubio L., Martinez B., Rodriguez A., Donovan D.M., Goetz F., Garcia P. (2013). The Phage Lytic Proteins from the *Staphylococcus aureus* Bacteriophage vB_SauS-phiIPLA88 Display Multiple Active Catalytic Domains and Do Not Trigger Staphylococcal Resistance. PLoS ONE.

[66] Gerstmans H., Rodriguez-Rubio L., Lavigne R., Briers Y. (2016). From endolysins to Artilysin(R)s: Novel enzyme-based approaches to kill drug-resistant bacteria. Biochem. Soc. Trans..

[67] Briers Y., Walmagh M., Van Puyenbroeck V., Cornelissen A., Cenens W., Aertsen A., Oliveira H., Azeredo J., Verween G., Pirnay J.-P. (2014). Engineered endolysin-based “Artilysins” to combat multidrug-resistant gram-negative pathogens. mBio.

